# The iron regulatory capability of the major protein participants in prevalent neurodegenerative disorders

**DOI:** 10.3389/fphar.2014.00081

**Published:** 2014-04-21

**Authors:** Bruce X. Wong, James A. Duce

**Affiliations:** ^1^Oxidation Biology Unit, The Florey Institute of Neuroscience and Mental Health, The University of MelbourneParkville, VIC, Australia; ^2^School of Molecular and Cellular Biology, Faculty of Biological Sciences, University of LeedsLeeds, UK

**Keywords:** Alzheimer’s disease, Parkinson’s disease, Huntington’s disease, prion disease, amyloid-β precursor protein, tau, α-synuclein, prion protein

## Abstract

As with most bioavailable transition metals, iron is essential for many metabolic processes required by the cell but when left unregulated is implicated as a potent source of reactive oxygen species. It is uncertain whether the brain’s evident vulnerability to reactive species-induced oxidative stress is caused by a reduced capability in cellular response or an increased metabolic activity. Either way, dys-regulated iron levels appear to be involved in oxidative stress provoked neurodegeneration. As in peripheral iron management, cells within the central nervous system tightly regulate iron homeostasis via responsive expression of select proteins required for iron flux, transport and storage. Recently proteins directly implicated in the most prevalent neurodegenerative diseases, such as amyloid-β precursor protein, tau, α-synuclein, prion protein and huntingtin, have been connected to neuronal iron homeostatic control. This suggests that disrupted expression, processing, or location of these proteins may result in a failure of their cellular iron homeostatic roles and augment the common underlying susceptibility to neuronal oxidative damage that is triggered in neurodegenerative disease.

## INTRODUCTION

The comparative ease in transition of valency states, most commonly between 2+ and 3+, over other metals makes iron one of the most useful metals in oxidative biology. In a cellular environment, over half of all enzymes are metalloproteins, a proportion of which establish complexes with Fe^3+^ and/or Fe^2+^ (Waldron et al., 2009). Within an aerobic environment iron is continuously redox cycling between Fe^3+^ and Fe^2+^ to produce reactive oxygen species (ROS). When liganded within proteins this cycling is safely guarded, however, when unprotected the ferrous form is prolific at producing very reactive and damaging hydroxyl radicals from O_2_ and H_2_O_2_ via Haber–Weiss and Fenton reactions (Fenton, 1894; Haber and Weiss, 1934). ROS production leads to DNA, lipid, and protein damage (Crichton and Ward, 2014); intrinsic factors to the increased oxidative stress and cellular damage in many neurodegenerative diseases.

## IRON WITHIN THE BRAIN

The brain not only requires iron for the usual fundamental metabolic process such as mitochondrial respiration and DNA synthesis, it is also essential for neurotransmitter synthesis and metabolism as well as myelin synthesis (Crichton, 2009). As iron mismanagement in either direction may severely damage the cell, homeostatic mechanisms have been evolutionarily incorporated to maintain optimal cell function (Wang and Pantopoulos, 2011). Unsurprisingly the proteins required to regulate cellular iron homeostasis in the brain are very similar to those used in the body’s periphery and rely on the two cytosolic labile iron pool sensors; iron response protein (IRP) 1 and 2, to bind to their respective iron regulatory elements in the untranslated mRNA (UTR) of iron responsive proteins (Muckenthaler et al., 2008). Binding to IRE’s located in the 5′-UTR prevent translation whereas IRP binding to 3′-UTR IRE’s protect mRNAs against nuclease degradation. This canonical *cis–trans* iron regulatory system increases IRP binding to the IRE when iron is required and allows: increased iron uptake through proteins such as transferrin receptor 1 (TfR1) and divalent metal ion transporter 1 (DMT1); impaired iron storage through ferritin (Ft); and reduced export via ferroportin (Fpn; Muckenthaler et al., 2008). When iron is in excess, the IRPs are no longer able to bind, allowing Ft and Fpn mRNA translation and increased mRNA degradation of TfR1 and DMT1.

Despite these common iron-regulated proteins being expressed within most cell types of the brain, the population of the cells present within the brain is diverse and dynamic in their function and requirement of iron. Therefore, differences in how each cell type regulates its iron is evident by the expression of proteins required for import, storage, and export of iron in neurons compared to neuroglia such as oligodendrocytes, astrocytes, and microglia. Circulatory iron transport in the brain is significantly carried out through association with small molecules such as citrate, ascorbate or ATP, however, the iron carrier transferrin is still present (Malecki et al., 1999; Ke and Qian, 2007). Despite this, neurons express TfR abundantly and are likely to acquire iron through the classical holotransferrin endocytosis followed by DMT1-mediated entrance of iron into the cytosol (Pelizzoni et al., 2012). In contrast, astrocytes and oligodendrocytes do not express TfR and therefore take up non-transferrin bound iron (NTBI) either through DMT1 (Lane et al., 2010) or some alternative routes involving the metal inward transporters; “transient receptor potential cation channel, subfamily C, member 6” (TRPC6; Mwanjewe and Grover, 2004; Giampa et al., 2007), “L-type voltage-dependent calcium channels” (L-VDCCs; Gaasch et al., 2007; Lockman et al., 2012) or “Zrt- and Irt-like proteins” (Zip8 and Zip14; Pinilla-Tenas et al., 2011; Jenkitkasemwong et al., 2012). Once inside, iron is mostly stored in Ft; however, abundance varies age-dependently between cell type with neurons typically containing the least and microglia containing the most (Benkovic and Connor, 1993). Iron may alternatively be stored by neuromelanin in select neuronal types, particularly those known to poorly express Ft such as the melanized dopaminergic neurons of the basal ganglia (Zecca et al., 2003; Snyder and Connor, 2009). Excess iron is exported from all cell types using Fpn, a unique iron efflux pore that is abundantly expressed in neurons, microglia, astrocytes, and oligodendrocytes (Song et al., 2010). However, spatiotemporal expression in neurons is variable (Moos and Rosengren Nielsen, 2006). A glycophosphatidylinositol-anchored form of ceruloplasmin (CP) expressed in astrocytes is known to facilitate iron efflux through Fpn (Jeong and David, 2003) and a similar role is proposed for hephaestin expressed in oligodendrocytes (Schulz et al., 2011) and amyloid-β precursor protein (APP) in neurons (Duce et al., 2010).

As well as each cell type’s ability to regulate its own iron content, a continual homeostatic interplay between neurons and the neuroglia is apparent as with most protein regulatory pathways. A good example of this is that despite there being limited presence of the soluble form of CP in the interstitial fluid (Singh et al., 2013), non-neuronal CP depletion causes age-dependent neuronal iron accumulation and cognitive impairment (Jeong and David, 2003). The full regulatory mechanisms of brain iron homeostasis has yet to be fully understood and are likely to include a number of redundant pathways for iron import, storage, and export that can be implemented in order to protect neurons from iron-induced oxidative stress.

Iron accumulation is evident in the aging brain from a range of animals including humans and whilst a heterogeneous distribution of iron is present within the brain, most regions have a continual increase in iron with lifespan (Aquino et al., 2009; Bilgic et al., 2012). Despite neuronal and neuroglial accumulation of iron with age, it has generally been considered not to associate with severe pathology, indicating that these cells are still capable of safely liganding the metal and guarding against oxidative stress-induced cellular damage. Evidence of this is shown with a correlative increase in the expression of the iron storage complex proteins Ft and neuromelanin with age (Connor et al., 1990; Zecca et al., 2001). To wholly understand the iron-induced pathology in neurodegenerative diseases, continued research is required to better understand the full homeostatic pathways for iron regulation in the brain.

## IRON IN NEURODEGENERATIVE DISEASE

Most of the brain’s iron is concentrated in the substantia nigra pars compacta (SN) and basal ganglia, together reaching comparable levels to that observed in the liver; a known peripheral repository of iron (Griffiths and Crossman, 1993; Haacke et al., 2005). Disrupted iron homeostasis focally in this region of the brain is evident in rare human disorders generally classed as “neurodegeneration with brain iron accumulation” (NBIA) disorders. The phenotypic symptoms of choreoathetosis, dystonia, parkinsonism, spasticity, and rigidity that are present in all forms of NBIA are predominantly associated with neuronal iron accumulation within this region of the brain [reviewed by Rouault (2013)]. The four most frequent subtypes have gene mutations in either; *PANK2* [pantothenate kinase-associated neurodegeneration (PKAN)], *PLA2G6* [PLA2G6-associated neurodegeneration (PLAN)], *C19orf12* [mitochondrial-membrane protein-associated neurodegeneration (MPAN)], or *WDR45* [beta-propeller protein-associated neurodegeneration (BPAN)]. A further five more rare NBIA disorders include Aceruloplasminemia (a deficiency in CP) and Neuroferritinopathy (a deficiency in Ft), and it is only in these two conditions that the functional mutation is in a known iron-regulated protein.

It has become increasingly evident that iron dyshomeostasis may not be pathologically restricted to NBIA disorders, but also a common underlying phenotype in more prevalent forms of neurodegenerative disease. The accumulation of iron in excess of that observed with age within these diseases may induce a variety of adverse effects, including increased oxidative stress, protein aggregation, mitochondrial dysfunction, and an imbalance in neurotransmitters; all of which are prevalent with neuropathology (Duce and Bush, 2010; Crichton and Ward, 2014).

The existence of a definitive correlation between brain iron homeostasis and neurotoxicity associated with these more prevalent forms of neurodegenerative disease still remains to be seen. Despite some studies suggesting that iron is non-specifically co-precipitated with the aggregated proteins pathologically observed with Alzheimer’s, Parkinson’s, Huntington’s, and prion diseases (Altamura and Muckenthaler, 2009), evidence described in this review provides support that iron has a role in the pathogenesis of these diseases and that previously known proteins associated with these disease pathologies are involved in neuronal iron homeostasis.

### PARKINSON’S DISEASE

Parkinson’s disease (PD) is the second most prevalent age-related neurodegenerative disease affecting 1–2% of the population over 65. It has received the most attention in explaining iron’s contribution to the pathogenesis of neurodegeneration. Patients present with motor dysfunction broadly arising from a loss of dopaminergic neurons within the pars compacta region of SN, while the SN reticulate is relatively unaffected. Elevated levels of total iron and a shift in the equilibrium of iron to the oxidized state within a region that already has a high level of iron in the brain is considered to contribute to the oxidative stress-induced neurotoxicity (Dexter et al., 1991; Halliwell, 1992; Wypijewska et al., 2010). However, more recently it has been observed that the combination of iron with dopamine is a greater risk factor than each element on their own, and that the SN pars compacta has a greater “iron-dopamine index” than other regions of the brain (Hare et al., 2014). Altered redox-active labile iron in PD is compounded by a loss of the buffering capacity of iron storage proteins; neuromelanin (Faucheux et al., 2003) and Ft (Connor et al., 1995) as well as iron-catalyzed aggregation of α-synuclein to form Lewy bodies in surviving neurons (Lotharius and Brundin, 2002). Contributing factors that can explain PD-increased iron accumulation are the elevated expression of the iron import transporter DMT1 (Salazar et al., 2008) and reduced expression of the iron export pore protein Fpn (Song et al., 2010) as well as CP ferroxidase activity (Olivieri et al., 2011; Ayton et al., 2012). *In vivo* imaging of iron by transcranial sonography (TCS) and T_2_^*^ -weighted magnetic resonance imaging (MRI) has strengthened the iron hypothesis of PD by illustrating a strong correlation for SN iron levels with disease severity and duration (Menke et al., 2009; Ulla et al., 2013). Typically, intraneuronal iron would be controlled by IRP1/2 response to the cytosolic labile iron pool. However, iron accumulation with an iron-regulated protein profile that correlates with decreased iron, infers a breakdown in the neuron’s iron regulatory system with PD. Support for this theory come from an inability for IRP to correctly respond in models of PD. Upregulation of IRP1/2 induces a downregulation in Fpn, thus exacerbating iron accumulation in a 6-hydroxydopamine model of PD (Song et al., 2010), but is unable to control Ft mRNA translation despite the elevated labile iron pool in PD (Hirsch, 2006).

### ALZHEIMER’S DISEASE

Accounting for 50–80% of all dementia cases, AD is the most common neurodegenerative disease of individuals over 65. The neuropathological hallmarks of AD are an accumulation of extracellular amyloid plaques comprising mainly of amyloid-β (Aβ), and the presence of intraneuronal neurofibrillary tangles that are comprised of hyperphosphorylated tau. Aβ is proteolytically derived from APP, a ubiquitously expressed type 1 transmembrane protein also predominantly expressed on the neuronal surface. A small number of AD cases who tend to have an earlier onset of disease are caused by autosomal dominance in familial mutations. These mutations are present within regions of APP or its γ-secretase cleavage proteins; presenilin 1 and 2, and promote the amyloidogenic processing of APP to increase Aβ generation. The trisomy mutation associated with Down’s syndrome also increases Aβ accumulation leading to early AD pathology and is considered to be caused by the increased copy number of APP that lies within chromosome 21.

While strong evidence suggests that Aβ is the principal cause of neurotoxicity and may be a significant contributor to synaptic dysfunction in AD (Roberts et al., 2012), iron accumulation in affected brain regions, as reported in both post mortem and MRI studies (Falangola et al., 2005; Jack et al., 2005; Antharam et al., 2012), may also be a factor in the increased oxidative stress observed in AD (Castellani et al., 2007). Hippocampal iron accumulation localized in neurofibrillary tangle-containing neurons and the neuritic processes surrounding senile plaques in AD (Quintana et al., 2006) correlates well with cognitive decline (Ding et al., 2009). Within the same regions, proteins regulated by iron are also altered whereby Ft is elevated (Grundke-Iqbal et al., 1990; Morris et al., 1994; Bouras et al., 1997; LeVine, 1997) and both CP expression and activity are lowered (Connor et al., 1993; Torsdottir et al., 2010). Despite these changes, transferrin expression levels are a little less clear with some evidence of a decrease (Connor et al., 1992) while later reports showing a localized increase within the frontal lobe (Loeffler et al., 1995). As previously suggested, the transport of iron by transferrin within the brain is minor and only required by select cells. This may account for the uncertainty in protein observations despite weak association with AD risk of the C2 variant to the *Tf* gene (Robson et al., 2004; Bertram et al., 2007). A known partner of Tf called HFE protein is also expressed in glia and neurons around neurofibrillary tangles and senile plaques of AD (Connor and Lee, 2006) and over the previous decade numerous genetic association studies have illustrated *HFE* gene mutations increase the risk of AD (Nandar and Connor, 2011). In particular the mutations H63D and C282Y (Sampietro et al., 2001; Pulliam et al., 2003; Blazquez et al., 2007) cause peripheral iron accumulation in AD and possibly has a link with the *APOE* gene. The HFE protein carrying the H63D mutation has also been shown to upregulate the phosphorylation of tau (Hall et al., 2010).

### HUNTINGTON’S DISEASE

Huntington’s disease (HD) is a progressive neurodegenerative disorder characterized by motor, psychiatric and cognitive disturbances that progress to dementia (The Huntington’s disease Collaborative Research Group, 1993). Prevalence in Europe, North America, and Australia is ~5.70 per 100,000 (Pringsheim et al., 2012). HD is caused by a dominant CAG expansion in the exon 1 encoded region of the *huntingtin* gene resulting in the expression of polyglutamine-expanded mutant huntingtin protein (The Huntington’s disease Collaborative Research Group, 1993). Similar to most neurodegenerative diseases and iron accumulative disorders, numerous mechanisms have been implicated in the pathogenesis of HD including oxidative stress (Browne and Beal, 1994), energetic dysfunction (Panov et al., 2002; Cui et al., 2006), transcriptional dysregulation (Nucifora et al., 2001; Dunah et al., 2002), and defective axonal transport (Trushina et al., 2004). Iron dysregulation occurs in human HD (Dexter et al., 1991; Rosas et al., 2012) and brain field map MRI values of gene-positive individuals have suggested that alterations of brain iron homeostasis occur before the onset of clinical signs (Rosas et al., 2012). Genetic mouse models of HD have also accurately recapitulated the elevated levels of brain iron (Fox et al., 2007; Chen et al., 2013). These findings were interpreted to indicate a compensatory response to iron stress occurring in HD striatum.

### PRION DISEASES

Prion diseases are a group of disorders whereby extreme cellular destruction leads to vacuolization and spongiosis of large areas of the brain. Within humans the most common form of prion disease is Creutzfeldt–Jacob disease (CJD) of which ~80% is sporadic cases. Despite being comparatively rare considering other forms of neurodegenerative disease, its high risk of infectivity both within and between species has prompted intense research into the disorder. Prion disease is now known to, at least in part, be caused by the conversion of the prion protein from its regular form (PrP^C^) into more of a β-sheeted isoform termed PrP-scrapie (PrP^Sc^; Prusiner, 1998; Aguzzi and Falsig, 2012). Little doubt remains that PrP^Sc^ is able to initiate infection when inoculated into a recipient animals brain (Wang et al., 2010) and that this is done by PrP^Sc^ autocatalyzing its conversion from PrP^C^. However, the mechanism by which PrP^Sc^ induces neurotoxicity remains to be fully elucidated as PrP^Sc^ levels poorly correlate with disease progression (Caughey and Baron, 2006).

Accumulation of redox-active iron, partly co-aggregated with Ft and in association with PrP^Sc^ plaques, has been reported in CJD brains (Petersen et al., 2005; Singh et al., 2009a, 2012). However, it appears that the increase in total iron may be biologically unavailable as the IRP response in the disease indicates iron deficiency. An increase in both Tf and its receptor as well as transcriptional changes with Ft and IRP1/2 are reported with Tf increase correlating with PrP^Sc^ levels (Kim et al., 2007; Singh et al., 2009a). In reflection to the prion diseased brain, cerebrospinal fluid (CSF) has decreased levels of Tf and increased total ferroxidase activity (Singh et al., 2011; Haldar et al., 2013). When used in combination these CSF markers of disease have an accuracy of 88.9% in detecting CJD over other forms of neurodegenerative disease (Haldar et al., 2013).

## THE ROLE OF PATHOLOGICAL PROTEINS IN IRON

As mentioned previously, a number of key iron homeostatic proteins such as CP and Ft, have been known for some time to cause NBIA disorders as well as be implicated in the more prevalent neurodegenerative diseases. However, recently a number of key proteins traditionally associated with the pathogenesis of the neurodegenerative diseases described above have also been implicated in an iron regulatory role in neurons. This has strengthened the argument that redox-active iron is a major facilitator of neurotoxicity in these diseases. Correlative studies on iron accumulation and altered pathology also support the theory that changes in iron homeostasis may be a feature in the early progress of the disease.

### AMYLOID-β PRECURSOR PROTEIN

As the name infers APP is the precursor of Aβ; the prevalent peptide found in senile plaques from a range of amyloidogenic diseases including AD. Proteolytic processing of APP from the neuron is predominantly through cell surface α-secretase cleavage followed by cleavage with the γ-secretase complex. This non-amyloidogenic processing of APP excludes Aβ production due to the α-secretase cleavage site residing within the Aβ peptide sequence. The alternative processing of APP through the amyloidogenic pathway requires β-secretase instead of α-secretase to produce Aβ as one of its products. The amyloidogenic processing of APP requires the protein to be endocytosed to allow optimal pH conditions for β-secretase cleavage. As yet it is not functionally clear as to why two intricate proteolytic pathways are required to cleave APP, however, the altered cellular location and function of cleaved products could be a likely reason.

Despite a reduced affinity compared to other transitional metals, iron binds Aβ and induces Aβ aggregation (Huang et al., 2004; Ha et al., 2007; Bousejra-ElGarah et al., 2011). This iron interaction is via His6, His13, and His14 of Aβ and is thought to be facilitated in a more reduced environment such as the brain due to the prevalence for the Fe^2+^ form of iron to bind Aβ (Bousejra-ElGarah et al., 2011). ROS generated through iron aggregated Aβ is toxic to neurons (Liu et al., 2011) and may partly contribute to the neurotoxicity present within the iron enriched environment around senile plaques (Meadowcroft et al., 2009; Gallagher et al., 2012). Evidence also supports the same histidine residues in Aβ binding to the iron center as well as porphyrin ring of heme (Atamna et al., 2009; Yuan and Gao, 2013). While the interaction with heme reduces Aβ aggregation (Zhao et al., 2013) it is unclear whether oligomeric Aβ, now considered to be the neurotoxic species within AD, are preferentially formed instead (Thiabaud et al., 2013). Heme binding to Aβ also restricts the bioavailability of regulatory heme and the complex formed has been shown to have peroxidase activity (Atamna and Boyle, 2006).

Translational regulation of APP through an IRE within the 5′UTR implies an interaction with iron status whereby increased cytosolic free iron levels translationally upregulate APP expression (Rogers et al., 2002). APP has recently been identified as a facilitator of neuronal iron efflux through an interaction with Fpn (Duce et al., 2010). While some controversy surrounds the exact mechanism of how APP is involved in the release of iron from the cell (Ebrahimi et al., 2012; Honarmand Ebrahimi et al., 2013), APP within a neuronal environment still appears to be essential to efflux iron (Duce et al., 2010; Wan et al., 2012). Depletion on APP in both cultured neurons and mouse models leads to intracellular iron retention that can be rescued upon the addition of APP to the extracellular environment (Duce et al., 2010) or by overexpression of cellular APP (Wan et al., 2012). Of significance, children suffering from Down’s syndrome that have an increased expression of APP have a reported high risk of iron deficiency and anemia (Dixon et al., 2010; Tenenbaum et al., 2011), however, further investigation is required to confirm whether this is due to an increase in APP facilitated iron efflux. As with Fpn, it appears that the surface presence of APP is essential for its role in iron efflux. When APP trafficking to the cell surface is impaired (Lei et al., 2012) or altered by processing through the amyloidogenic pathway, as with the AD-associated familial mutation in APP, iron accumulation arises (Wan et al., 2011).

### TAU

Hyperphosphorylated tau has mostly been recognized as the principal component of neurofibrillary tangles, a pathological hallmark in a number of neurodegenerative disorders including AD. Various repeat motifs on tau are known to bind iron in a pH- and stoichiometric-dependent manner that results in the promotion of phosphorylation and aggregation of the protein (Ma et al., 2006; Malm et al., 2007; Zhou et al., 2007). While the affinity for iron within a physiological environment has yet to be established for tau, binding of Fe^2+^ appears to preferentially induce phosphorylation of tau (Lovell et al., 2004; Chan and Shea, 2006) despite Fe^3+^ being the favored state in causing aggregation of tau once it has been hyperphosphorylated (Yamamoto et al., 2002; Amit et al., 2008).

Recently, it has been identified that tau may be required in the iron-modulatory role of APP (Lei et al., 2012). Mice deficient in tau have neuronal iron accumulation that can be reduced in primary cultures by the extracellular addition of APP or moderate chelators such as clioquinol (Lei et al., 2012). Tau has been implicated in axonal trafficking of proteins including APP (Islam and Levy, 1997) and it is proposed that impaired trafficking of APP to the cell surface in tau^-/-^ neurons restricts APP’s ability to facilitate iron efflux through Fpn leading to intracellular iron accumulation (Lei et al., 2012).

### α-SYNUCLEIN

Variance in α-synuclein is sufficient to cause PD in humans and animal models suggesting a central role in PD pathogenesis (Hardy, 2010). This is apparent through several observations; the overexpression of wild-type α-synuclein through gene duplication is sufficient to cause parkinsonian symptoms (Singleton et al., 2003; Chartier-Harlin et al., 2004; Fuchs et al., 2007); the majority of familial cases of PD are associated with mutations in α- synuclein (Polymeropoulos et al., 1997; Kruger et al., 1998; Zarranz et al., 2004; Hardy, 2010); and aggregated α-synuclein is a core protein found in Lewy bodies. It has been proposed that this ubiquitously expressed protein is involved in synaptic vesicle formation however, it is currently not understood why the dopaminergic neurons of the SN that are targeted in PD are more susceptible to α-synuclein aggregation and toxicity. One theory as to the vulnerability of the SN is the high levels of iron within the region that can augment α-synuclein aggregation. Iron has been shown to bind to the C-terminal region of α-synuclein and under oxidizing conditions, such as that provided in dopamine’s presence, denatures the protein and promotes further aggregation (Cappai et al., 2005; Binolfi et al., 2006; Bharathi et al., 2007). Redox-active iron is detected in association with α-synuclein aggregates in Lewy bodies (Castellani et al., 2000), a phenotype that is likely to cyclically promote iron-mediated oxidation and exacerbate the aggregation of α-synuclein as well as the other proteins co-aggregated within the Lewy body (Giasson et al., 2000).

As with APP a strong indication of the importance of α-synuclein in the regulation of neuronal iron was through the identification of an iron-response element in its 5′UTR that is required to increase translation when intraneuronal iron is high (Friedlich et al., 2007; Febbraro et al., 2012). In support of an iron-associated role of α-synuclein, it has recently been identified to modulate cellular iron homeostasis through its ability to reduce Fe^3+^ into the biologically active Fe^2+^ form (Davies et al., 2011). In accordance with its ferrireductase activity, α-synuclein has a greater affinity for Fe^3+^ (Rouault and Tong, 2005) and within a normal physiological environment such as the SN may provide a consistent supply of Fe^2+^ for neuronal metabolic processes such as enzymatic synthesis of neurotransmitters.

### PRION PROTEIN

As well as the significant role iron has in prion disease pathogenesis through PrP^Sc^ – Ft generated ROS that was described above, recent reports have also suggested that PrP^C^ has a normal physiological role in iron uptake. Cell surface presentation of PrP^C^ is required for this function and it appears the copper binding octapeptide repeat region of the protein may have ferrireductase activity (Singh et al., 2013). When the non-modified form of PrP^C^ is overexpressed in cultured cells, iron uptake, and storage is increased (Singh et al., 2009c, 2013) and similarly when expression is depleted by gene knockout in mouse models, tissue iron deficiency correlates with changes to proteins responsive to iron (Singh et al., 2009b). Intriguingly, a familial mutation in PrP^C^ (P102L) classically associated with the prion disorder called Gerstmann–Sträussler–Scheinker (GSS) disease is shown to increase ferrireductase activity and increase levels of intracellular labile iron (Singh et al., 2013). Accumulating evidence now suggests that PrP^C^ may have a role in the transferrin and NTBI import into the cell similar to DMT1 or Zip14. Of note, PrP^C^ bears a phylogenetic relationship to the ZIP family (Schmitt-Ulms et al., 2009) and has recently been implicated in neuronal zinc import when complexed to NMDA receptors (Watt et al., 2012). Similar to the recent identification that Zip14 is able to transport iron as well as zinc, it is worth noting that the NMDA- PrP^C^ complex involved in zinc import may also be implicated in neuronal iron import under certain conditions.

### HUNTINGTIN

Mutant huntingtin protein aggregates to form inclusion bodies that represent a pathological hallmark of HD. As with most aggregated protein structures formed in neurodegenerative disease, these bodies bind iron and act as centers of oxidative stress with large amounts of oxidized protein present (Firdaus et al., 2006). Genetic mouse models of HD that transgenically overexpress mutant huntingtin accurately recapitulate the elevated levels of brain iron in the disease (Fox et al., 2007; Chen et al., 2013) and huntingtin knockdown in zebra fish models result in an iron deficiency phenotype (Hilditch-Maguire et al., 2000; Lumsden et al., 2007; Henshall et al., 2009). This suggests that huntingtin is not only regulated by iron but also involved in iron homeostasis. However, iron does not interact directly with N-terminal huntingtin fragments (Fox et al., 2007; Chen et al., 2013) indicating that the effect of huntingtin on iron may mediate downstream influences on iron homeostatic pathways.

## CONCLUSION; BRAIN IRON DYSHOMEOSTASIS AS A THERAPEUTIC TARGET

With increasing evidence indicating that iron dyshomeostasis may be a mechanism of exacerbating disease pathology in these more prevalent forms of neurodegenerative disease, there is an escalating realization for its use as a viable target for new therapeutic design. Instrumental work carried out on therapeutic design in the body’s periphery (Higgs et al., 2012; Zhou et al., 2012) has increasingly been implemented to investigate their value at restoring iron homeostasis within the brain (Zecca et al., 2004; Badrick and Jones, 2011; Zorzi et al., 2012). However, a significant hurdle in the use of these drugs has been the relative impermeability to the blood brain barrier for some of the more effective peripheral tissue therapeutics and the necessity to target iron in brain rather than the periphery. This barrier is required to isolate and protect the brain from the peripheral circulatory system and transport of drugs across must either occur via active transport using receptors or small lipid soluble molecules that can diffuse across the cellular plasma membrane [for review see (Zheng and Monnot, 2012)].

Iron selective chelators such as desferrioxamine have had limited success in the brain when administered peripherally, largely due to their size and impermeability of the blood brain barrier (Richardson, 2004). However, a number of smaller molecular compounds with varying affinity for iron such as deferiprone, deferasirox, and clioquinol as well as their derivatives, have had promising outcomes in preclinical trials on models of neurodegenerative disease (Kaur et al., 2003; Atamna and Frey, 2004; Molina-Holgado et al., 2008; Rival et al., 2009; Prasanthi et al., 2012). Recently there have been a series of reviews comprehensively discussing preclinical studies on metal affinity compounds [for example Duce and Bush (2010), Ward et al. (2012), Weinreb et al. (2013)]. Deferiprone has already been clinically approved for the peripheral iron overload disorder thalassemia, and used in other neurodegenerative diseases such as Friedreich’s ataxia (Boddaert et al., 2007). A recent pilot clinical trial to test for safety and efficacy indicated 6-months deferiprone treatment of early stage PD patients decreased motor handicap progression (as measured by Unified PD Rating Scale) as well as SN iron deposition (as measured by R2^*^MRI; Devos et al., 2014) and a further trial is currently underway (Clinical trial #’s NCT01539837). Similarly, clinical trials in AD patients using a clioquinol derivative called PBT2 that has weaker affinity for iron than other translational metals such as copper and zinc, reduced Aβ in CSF as well as improved executive cognitive function (Lannfelt et al., 2008) and is currently in a further clinical trial on AD and HD patients (Clinical trial #’s NCT00471211 and NCT01590888).

While metal affinity compounds have been the focus of most iron therapeutic research in the past decade (Duce and Bush, 2010; Badrick and Jones, 2011; Roberts et al., 2012; Zorzi et al., 2012), it is evident that care must be taken so as to prevent excessive binding which could result in removal of too much iron from the neuronal environment as the reduction in levels below that required for normal physiological maintenance could be just as detrimental to survival (as indicated in anemia). Inaccurate administrative dose of a chelator may therefore only compound the disease phenotype in which they are being used to ease or alter the disease phenotype toward an iron deficiency-like pathology that is just as harmful to the patient. Upon better understanding of the brain’s capacity in regulating iron homeostasis both within and between cell types perhaps an alternative approach in the future may be to utilize the brain’s own iron homeostatic system to restore the balance of iron. In so doing, recompartmentalizing iron to areas within the brain that have a better capability to cope with oxidative stress-induced by redox-active iron may go a long way toward alleviating the common underlying defects that occur in these more prevalent neurodegenerative diseases.

## Conflict of Interest Statement

The authors declare that the research was conducted in the absence of any commercial or financial relationships that could be construed as a potential conflict of interest.
